# The role of urea-induced osmotic diuresis and hypernatremia in a critically ill patient: case report and literature review

**DOI:** 10.1590/2175-8239-JBN-2018-0226

**Published:** 2019-04-25

**Authors:** Jesiree Iglésias Quadros Distenhreft, Júlia Guasti Pinto Vianna, Gabriela S. Scopel, Jayme Mendonça Ramos, Antonio Carlos Seguro, Weverton Machado Luchi

**Affiliations:** 1 Universidade Federal do Espírito Santo, Serviço de Nefrologia, Hospital Universitário Cassiano Antonio Moraes, Vitória, ES, Brasil.; 2 Universidade de São Paulo, Laboratório de Investigação Médica, Hospital das Clínicas da Faculdade de Medicina da São Paulo, São Paulo, Brasil.

**Keywords:** Diuresis, Hypernatremia, Urea, Critical Care, Diurese, Hipernatremia, Ureia, Cuidados Críticos

## Abstract

Hypernatremia is a common electrolyte problem at the intensive care setting, with a prevalence that can reach up to 25%. It is associated with a longer hospital stay and is an independent risk factor for mortality. We report a case of hypernatremia of multifactorial origin in the intensive care setting, emphasizing the role of osmotic diuresis due to excessive urea generation, an underdiagnosed and a not well-known cause of hypernatremia. This scenario may occur in patients using high doses of corticosteroids, with gastrointestinal bleeding, under diets and hyperprotein supplements, and with hypercatabolism, especially during the recovery phase of renal injury. Through the present teaching case, we discuss a clinical approach to the diagnosis of urea-induced osmotic diuresis and hypernatremia, highlighting the utility of the electrolyte-free water clearance concept in understanding the development of hypernatremia.

## Resumo

A hipernatremia é um distúrbio eletrolítico comum no ambiente de terapia intensiva, com uma prevalência que pode chegar a 25%. Está associada a maior tempo de internação hospitalar e é um fator de risco independente para a mortalidade. Este relato ilustra um caso de hipernatremia de origem multifatorial no ambiente de terapia intensiva. Destacaremos o papel da diurese osmótica por geração excessiva de ureia, uma causa de hipernatremia pouco conhecida e subdiagnosticada. Este cenário pode estar presente em pacientes em uso de elevadas doses de corticoides, com sangramento gastrointestinal, em uso de dietas e suplementos hiperproteicos e estado de hipercatabolismo, especialmente durante a fase de recuperação de injúria renal. A seguir, discutiremos uma abordagem clínica para o diagnóstico da hipernatremia secundária à diurese osmótica induzida por ureia, destacando a importância do conceito de clearance de água livre de eletrólitos nesse contexto.

## INTRODUCTION

Hypernatremia is a common electrolyte problem at the intensive care setting, with a prevalence that can reach up to 25%. It is associated with a longer hospital stay and is an independent risk factor for mortality.^1^ The condition reflects a water deficit in relation to the amount of sodium in the body, which may occur due to electrolyte-free water loss and/or sodium gain. The loss of free water secondary to osmotic diuresis often results from high concentrations of glucose or urea in the urine or from the use of mannitol.^2^ In critically ill patients, high protein supply, hypercatabolism, gastrointestinal bleeding, and high doses of corticosteroids may promote excessive urea generation, osmotic diuresis, and consequently hypernatremia.^3^ The following case report illustrates the diagnostic approach of hypernatremia associated with osmotic diuresis secondary to the excessive urea generation, focusing on the importance of the calculation of electrolyte-free water clearance in this context.

## CASE REPORT

### Clinical History and Initial Laboratory Tests

A 72-year-old woman with a previous medical history of hypertension, type 2 diabetes mellitus, and heart failure, was admitted to the emergency room with hypertensive acute pulmonary edema (blood pressure: 250/150 mmHg), requiring invasive mechanical ventilation. Electrocardiogram showed left bundle branch block, but markers for myocardial necrosis were negative and cardiac catheterization did not show significant obstructions. Laboratory tests at admission did not demonstrate noteworthy alterations.

On the fourth day of hospitalization, she was extubated, but presented severe laryngospasm refractory to clinical measures, as corticoid therapy, needing reintubation. Subsequently, the patient developed ventilator-associated pneumonia and serum sodium level progressively increased, reaching 165 mEq/L (Table 1). Treatment with 0.45% NaCl solution and free water through nasoenteric feeding tube were prescribed, without improvement of natremia. In addition, due to signs of congestion on chest radiography, peripheral edema and oliguria, 40 mg / day of intravenous furosemide was started. Then, during the ventilator weaning process, physical examination evidenced right hemiparesis. Cranial computed tomography revealed a hemorrhagic stroke in the left middle cerebral artery region. After extubation, the patient remained with aphasia and dysphagia, persisting with the need of enteral diet. Table 1 summarizes the evolution of laboratorial parameters and relevant clinical data.

**Table 1:** Evolution of laboratory and clinical parameters during hospitalization*

Blood	On admission	Day 4^a^	Day 6 ^b^	Day 12 ^c^	Day 14 ^d^ ^,^ ^e^	Day 19	Reference Range
Creatinine (mg/dL)	0.8	0.79	0.86	1.88	1.53	0.59	0.7 - 1.2
Urea (mg/dL)	25	61	80	206	239	49	10 - 25
Sodium (P_Na_) (mEq/L)	139	141	150	153	165	141	135 - 145
Chlorine (mEq/L)	102	101	106	101	117	100	98 - 107
Potassium (mEq/L)	3.9	4.2	3.3	3.9	3.4	4.04	3.5 - 5
Bicarbonate (mEq/L)	24.7	25.2	30	36.5	24	22	22 - 24
Glucose (mg/dL)	130	139	181	247	270	208	<140
Osmolarity (P_Osm_) (mOsm/L)					345		275 - 295
**Urine Output (24-hour)**							
Urine Volume (U_Vol_) (mL)		800	1,500	2,055	2,025	2,500	< 3,500
Creatinine (g)					1.05 g (51.63mg/dL)		0.7 - 1.3
Urea (g)					48.9 g (2,413 mg/dL)	24.6g (984 mg/dL)	15 - 35
Sodium (U_Na_) (mEq/L)					22	68	40 - 220
Potassium (U_K_) (mEq/L)					62	25	15 - 125
Glucose (g)			Negative**		0.072 (4 mg/dL)		< 0.5
Osmolarity (U_Osm_) (mOsm/L)					570	350	50 – 1,200
Measured urine osmoles							
Total Osmoles					1,154	875	600 - 800
Osmoles from Urea					814	410	350 - 450
Osmoles from Na + K					340	465	300 - 350

^a^ Corticosteroid and furosemide start;

^b^ Hiperprotein diet start;

^c^ Suspension of furosemide;

^d^ Nephrology visit day;

^e^ Corticosteroid suspension and diet replacement for normoprotein.

* Calculation and formulas used in this Table are shown using the example of nephrology visit day, represented in Box 1.

** Negative glucose by dipstick test.

### Additional investigation

On the 14th day of hospitalization, the nephrology team was assessed for additional investigation of hypernatremia. The patient was clinically hypervolemic, tending to hypertension, with an accumulated positive fluid balance despite diarrhea and febrile condition, and urine volume (UV) around two liters per day. She was still receiving high doses of intravenous corticosteroid (methylprednisolone 75 mg 8/8h), initially prescribed due to laryngospasm. Furosemide had been discontinued on the 12th day of hospitalization due to worsening renal function and hypernatremia. The enteral diet characteristics were: diet for diabetic patient, volume per day = 1,500 mL, hyperproteic (75 g/L), with osmolarity of 530 mOsm/L, and sodium concentration of 17 mEq/L. According to the estimated weight of 60 kg, she was receiving 1.8 g/kg of protein.

As depicted in Table 1, additional laboratory tests of plasma and 24-hour urine were performed at the nephrology visit day, and the following calculations were obtained: free water clearance (CH_2_O) = - 1,320 mL; electrolyte-free water clearance (C_e_H_2_O) = + 957 mL; total urine osmoles = 1,154 mOsm; urine osmoles generated by urea = 814 mOsm; and urine osmoles generated by Na + K = 340 mOsm. Based on these results, the diagnosis of hypernatremia was established. Box 1 describes the formulas and calculations used in this case.

**BOX 1:** Calculation and formulas used in nephrology visit day

**1. Effective Plasma Osmolarity:**		
2 [P_Na_ (mEq/L)] + [glucose (mg/dL)/18] 2 [165] + [270/18] = 345 mOsm/kg		
**2. Urine Osmolarity:**		
2 [U_Na_ (mEq/L) + U_K_ (mEq/L)] + [Urine urea (mg/dL)/6] + [Urine glucose (mg/dL)/18] 2 [22 + 62] + 2413/6 + 4/18 = 570 mOsm/kg		
**3. Daily excretion of urine osmoles (calculated):**		
**Total**	**From Urea**	**From Na + K**
Calculated U_Osm_ X 24h U_Vol_ 570 mOsm/kg X 2,025 L 1,154 osmoles	[Urine urea (mg/dL)/6] X 24h U_Vol_ 402 mOsm/kg X 2,025 L 814 osmoles	2 [UNa (mEq/L) + U_K_ (mEq/L)] X 24h U_Vol_ 168 mOsm/kg X 2,025 L 340 osmoles
**4. Free Water Clearance (CH_2_O):**		
U_Vol_ (mL) x (1 - U_Osm_/P_Osm_) 2.025 x (1 – 570/345) = - 1320 mL		
**5. Electrolyte Free Water Clearance (C_e_H_2_O):**		
$U_{\mathrm{Vol}}\left(\mathrm{mL}\right)x\left[\frac{1-\left(U_{\mathrm{Na}}\left(\mathrm{mEq}/L\right)+U_{K}\right)}{P_{\mathrm{Na}}\left(\mathrm{mEq}/L\right)}\frac{\left(\mathrm{mEq}/L\right)}{\;}\right]=\frac{2.025x}{\;}\frac{\left[1-\left(22+62\right)\right]}{165}\;=+957\mathrm{mL}$		

### Diagnosis

Hypernatremia induced by osmotic diuresis secondary to excessive urea generation.

### Clinical follow-up

After diagnosis, the initial approach was the reduction of the protein supply of the enteral diet to 1.0 g/kg, as well as tapering of corticosteroid doses. Five days from the establishment of these measures, urinary urea decreased to 24.6 g per day, corresponding for 410 mOsm of the total of 875 mOsm in the urine, correcting the previous osmotic effect induced by excessive urea generation. Likewise, progressive decrease on plasma urea and sodium levels were observed (Table 1).

## DISCUSSION

This report illustrates a case of hypernatremia of multifactorial etiology in the intensive care unit (ICU). Although furosemide has an initial role in elevating serum sodium levels, we will highlight the role of osmotic diuresis by excessive generation of urea, an underdiagnosed and a not well-known cause of hypernatremia. Although prevalence data in the literature are scarce, the study by Lindner G et al. demonstrated that osmotic diuresis secondary to urea was the causative factor in 10% of the cases that evolved to hypernatremia in the ICU.^3^ Below, we discuss a clinical approach to the diagnosis of urea-induced osmotic diuresis and hypernatremia, emphasizing the utility of physiological concepts on the understanding of the development of hypernatremia.

**Figure 1:**
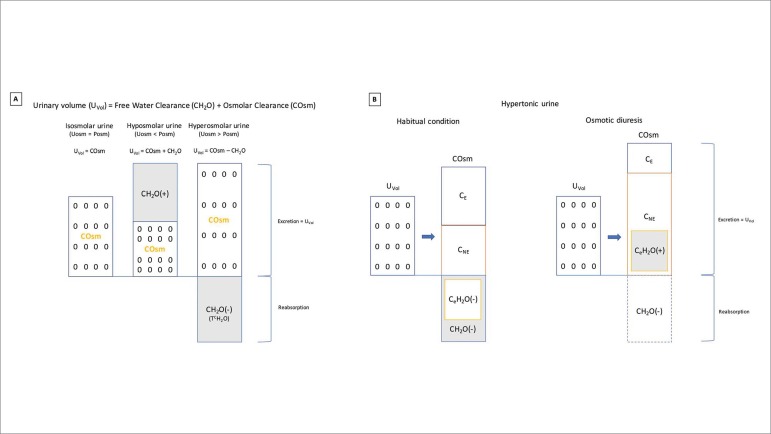
**A**. Urinary volume (U_VOL_) composition and its relation with osmolar clearance (COsm), free-water clearance (CH_2_O), and electrolyte-free water clearance (C_e_H_2_O) in situations with different urinary tonicity: isosmolar, hypertonic, and hypotonic. **B.** Usual situation during a hypertonic urine production: CH_2_O is negative, indicating that the body is saving water. It occurs because of the increase of solute-free water reabsorption in the collecting duct by antidiuretic hormone, referred to as T^C^H_2_O. Note that C_e_H_2_O is also negative, demonstrating that most of the solutes (osmoles) of the urine are electrolytes, Na^+^ and K^+^ (C_E_). Inversely, in an osmotic diuresis scenario, the calculation of CH_2_O is negative, but C_e_H_2_O is positive. It displays that most of the solutes are non-electrolyte (C_NE_>C_E_) and most of the urinary volume excreting these solutes, instead of the electrolyte solutes, characterizing water loss and not water retention. C_E_ = electrolyte clearance; C_NE_ = nonelectrolyte clearance; Uosm = urine osmolarity; Posm = plasma osmolarity.

As shown in Figure 1A, when the UV collected in 24 hours is dismembered, part of the volume is designated to the solute clearance (osmolar) and part to the water clearance. Osmolar clearance (COsm) is defined as the part of UV necessary to excrete all solutes, electrolytes (sodium, potassium, etc) and non-electrolytes (urea, creatinine, glucose, etc), in a supposedly isotonic urine compared to plasma. On the other hand, the portion of UV free from all solutes represents the free water clearance (CH_2_O), which can also be defined as the amount of water that needs to be added or removed from COsm in order to complete the total UV measured in 24 hours. In addition, the part of UV that is free only from the solutes composed by electrolytes is called electrolyte-free water clearance (C_e_H_2_O).^3^^,^ 4

In this scenario, the CH_2_O modulation is specially determined by the antidiuretic hormone (ADH), which in turn varies according to the natremia. Usually, in the presence of increased serum osmolarity, the CH_2_O becomes negative due to ADH increase, making the urine hyperosmolar compared to the plasma and indicating that the body is saving water. Conversely, the reduction of serum osmolarity makes CH_2_O positive, and urine osmolarity (UOsm) turns lower than the plasmatic. This indicates that the body is losing water. Nevertheless, as discussed further, the C_e_H_2_O is what in fact accounts for water loss or retention in face of alterations in plasma sodium.^5^^,^ 6

Formulas of urine and plasma osmolarity predominantly include sodium, potassium, glucose, and urea concentrations (Box 1). However, considering that urea does not have electric charge and may freely move from intracellular to extracellular fluid, it cannot generate an effective osmotic gradient. Thus, even with high serum levels, urea causes little or no increase in effective plasma osmolarity and ADH release.^7^ In contrast, although urea is partially reabsorbed on the proximal tubule, high concentrations of this solute in this segment might induce osmotic properties, as it behaves as a non-permeable solute. Urea participates in the urinary concentration since it accumulates on the medullary interstitium, reinforcing the countercurrent mechanism for water reabsorption. Nonetheless, the optimal proportion between the osmolarity generated in the urine by urea and by non-urea solutes is of 0.2 to 1.2. Above this interval, the excessive urea excretion will have little or no influence on the medullary interstitium concentration. Consequently, it remains on the tubules and implies an osmotic gradient, which is responsible for free-electrolyte water loss.^6^^,^ 8 This mechanism originates hypernatremia. In clinical practice, besides the ratio between the UOsm generated by urea and by non-urea solutes >1.2, the urinary concentration of urea greater than 250 mmol/L (> 1,500 mg/dL) also suggests the presence of the osmotic effect.^5^

**Figure 2:**
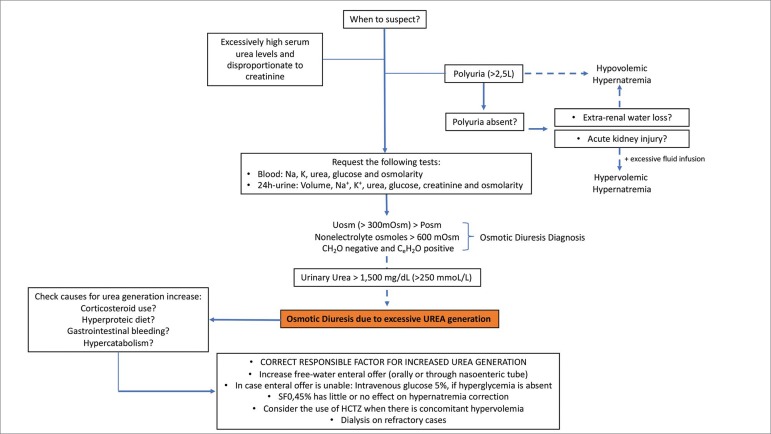
Algorithm illustrating the diagnostic approach for urea-induced hypernatremia.

In addition to the excessive generation of urea, osmotic diuresis may be present in the following situations: glycosuria (> 250 mmol/L or > 4.5 g/dL), use of mannitol, and during the recovery phase of an acute kidney injury (AKI).^4^ In these situations, UOsm will be higher than plasma osmolarity due to the excess of non-electrolyte solutes. Since the calculation of the UOsm considers all the solutes (electrolyte and nonelectrolyte) on its formula, the CH_2_O will be negative, mistakenly suggesting water retention (Box 1 and Figure 1B). However, the C_e_H_2_O does not consider urea and other nonelectrolyte solutes on its formula and therefore is more accurate than the CH_2_O to predict the occurrence of water loss or retention.^3^^,^ 4 Additionally, we can also infer the presence of free water loss when the sum of Na^+^ + K^+^ concentrations in urine is lower than Na^+^ concentration in plasma [(U_Na+K_)/(P_Na_) < 1] in patients with hypernatremia. Briefly, osmotic diuresis may be suspected when UOsm is > 300 mOsm/L (or higher than serum osmolarity) in the presence of polyuria (usually > 2.5 L/24h) associated with a negative CH_2_O and a positive C_e_H_2_O.^9^^,^ 11 The confirmation is given when the nonelectrolyte solutes excretion rate is higher than 600 mOsm/24h (Figure 2). Interestingly, the UV may be < 2.5 L/24h when there is a concomitant AKI or an important extra renal water loss.^5^^,^ 9

In the reported case, the urinary urea concentration was 2,413 mg/dL, responsible for 402 mOsm/L out of 570 mOsm/L of UOsm (urea generated osmolarity / non-urea solutes ratio = 2.39), emphasizing the urea contribution on the total amount of osmoles in the urine (814 mOsm out of the total of 1,154 mOsm) (Table 1). Although the patient had hyperglycemia, the glycosuria was negligible and was not the responsible factor for osmotic diuresis. The elevated plasmatic urea levels present in this case may be related to the use of high doses of corticoid, high-protein diet, and hypercatabolic state associated with infectious disease. If only the generation of urea due to the hyperproteic diet was considered, we would have a urea production of approximately 36 g ((34% of the 108 g of protein offered in the diet) of the total 48.9 g excreted in the urine (Table 1), indicating that there are other associated factors contributing to the generation of urea.

From the above, the patient presented a UOsm higher than the plasmatic (570 vs 345 mOsm/L) and a negative CH_2_O of 1,320 mL, suggesting water retention during hypernatremia. However, the positive C_e_H_2_O value of 957 mL/day, and (U_22 + 62_)/(P_165_) <1, indicated that there was free water loss. The positive C_e_H_2_O allowed the inference that the urine is actually diluted and not concentrated in relation to plasma, since the electrolytes are responsible for only 168 mOsm/L out of the 570 mOsm/L of UOsm. Urine hyperosmolarity occurs due to urea. Therefore, water retention is not the case, but free water loss.

It is important to mention that the increase in serum sodium levels observed in the first days of hospitalization (D6) was not related to excessive urea generation but to the use of furosemide. This diuretic drug was prescribed on the fourth day considering pulmonary congestion, oliguria, and lower limb edema, in the context of extubation failure. However, it was responsible for the metabolic alkalosis and hypokalemia as observed in Table 1. Although the patient maintained a positive accumulated water balance, furosemide was suspended due to the declining renal function. Subsequently, hypernatremia continued to worsen as a consequence of urea-induced osmotic diuresis. Usually, this is a cause of hypernatremia that tends to occur later in the course of hospitalization, since it requires a longer time for the excessive production of urea triggered by the factors described above.

Several factors were added throughout the hospitalization of our patient that resulted in the state of hypervolemic hypernatremia. Among them: hydrosaline overload, sodium retention by corticosteroid, oliguria secondary to AKI, use of furosemide, and the greater loss of water relative to sodium loss during osmotic diuresis. Hypervolemic hypernatremia has been reported in ICU during the renal function recovery phase in patients initially exposed to severe hydrosaline overload. At this point, despite progressive glomerular filtration rate improvement, tubular urea handling still remains compromised, and the release of an accumulated surplus of urea favors osmotic diuresis.^10^ Nevertheless, it was during the plasma creatinine decrease (D12->D14, Table 1) that the serum sodium level reached the highest value.

In addition, the polyuria expected during osmotic diuresis phase was not evident in our case probably as a result of extra renal water loss by diarrhea and febrile state, and by AKI. Conversely, in the absence of AKI and/or hydrosaline overload preceding or concomitant to the excessive urea generation, hypernatremia will evolve with hypovolemia (Figure 2). Furthermore, because of the disproportionate increase of plasma urea in relation to creatinine, the clinical presentation will appear to be a pre-renal AKI pattern.

**Figure 3:**
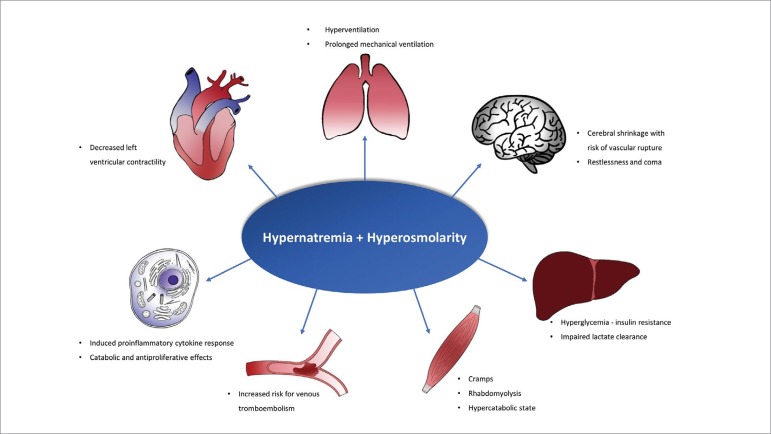
Representation of the multiple consequences of hypernatremia and hyperosmolarity state on the body functions. Adapted from Lindner G et al.^11^

Hypernatremia and the associated hyperosmolar state lead to multiple effects on the body functions that are summarized in Figure 3.^10^^,^ 11 In the central nervous system, hyperosmolarity displaces free water from the intracellular to the extracellular space, shrinking the brain cells, which can cause vascular rupture and which may have contributed to the development of stroke in our patient. The treatment priority of hypernatremia induced by osmotic diuresis is to remove the causal factor and to increase pure-water offering (Figure 2). It is important to mention that the use of a hypotonic solution at 0.45% NaCl (Osm: 154 mOsm/L) will have little or no effect on sodium decrease, because the osmolarity of this solution will be similar to the fraction of the osmolarity determined by the electrolytes in the urine (in our case, 168 mOsm/L).^3^ When fluid overload is present, the use of thiazide diuretics may contribute to the treatment. Thiazide increases urine volume, but also causes a greater increment in total solute excretion, and therefore instead of increasing CH_2_O, it reduces C_e_H_2_O.^12^ When renal function is in recovery phase, the resolution of hypernatremia occurs in a few days. Otherwise, dialysis may be required.

In summary, the deleterious effects of hypernatremia reinforce the necessity of a judicious analysis of the underlying mechanisms involved on its pathogenesis. Osmotic diuresis due to excessive urea generation must be one of the differential diagnosis of the causes of hypernatremia at the ICU, since critical patients are susceptible to the triggering factors as use of hyperproteic diets, hypercatabolism, use of high doses of corticosteroids, and gastrointestinal bleeding, especially in the context of AKI recovery. The recognition of C_e_H_2_O is the cornerstone for this diagnosis.
